# m^6^A regulator expression profile predicts the prognosis, benefit of adjuvant chemotherapy, and response to anti-PD-1 immunotherapy in patients with small-cell lung cancer

**DOI:** 10.1186/s12916-021-02148-5

**Published:** 2021-11-22

**Authors:** Zhihui Zhang, Chaoqi Zhang, Yuejun Luo, Peng Wu, Guochao Zhang, Qingpeng Zeng, Lide Wang, Zhaoyang Yang, Liyan Xue, Bo Zheng, Hua Zeng, Fengwei Tan, Qi Xue, Shugeng Gao, Nan Sun, Jie He

**Affiliations:** 1 Department of Thoracic Surgery, National Cancer Center/National Clinical Research Center for Cancer/Cancer Hospital, Chinese Academy of Medical Sciences and Peking Union Medical College, Beijing, 100021 China; 2 Department of Pathology, National Cancer Center/National Clinical Research Center for Cancer/Cancer Hospital, Chinese Academy of Medical Sciences and Peking Union Medical College, Beijing, 100021 China

**Keywords:** m^6^A regulators, Small cell lung cancer, Chemotherapy, Immunotherapy, Individualized medicine

## Abstract

**Background:**

Small cell lung cancer (SCLC) is lethal and possesses limited therapeutic options. Platinum-based chemotherapy—with or without immune checkpoint inhibitors (anti-PDs)—is the current first-line therapy for SCLCs; however, its associated outcomes are heterogeneous. *N*^6^-methyladenosine (m^6^A) is a novel and decisive factor in tumour progression, chemotherapy resistance, and immunotherapy response. However, m^6^A modification in SCLC remains poorly understood.

**Methods:**

We systematically explored the molecular features and clinical significance of m^6^A regulators in SCLC. We then constructed an m^6^A regulator-based prognostic signature (m^6^A score) based on our examination of 256 cases with limited-stage SCLC (LS-SCLC) from three different cohorts—including an independent cohort that contained 150 cases with qPCR data. We additionally evaluated the relationships between the m^6^A score and adjuvant chemotherapy (ACT) benefits and the patients’ responses to anti-PD-1 treatment. Immunohistochemical (IHC) staining and the HALO digital pathological platform were used to calculate CD8+ T cell density.

**Results:**

We observed abnormal somatic mutations and expressions of m^6^A regulators. Using the LASSO Cox model, a five-regulator-based (*G3BP1*, *METTL5*, *ALKBH5*, *IGF2BP3*, and *RBM15B*) m^6^A score was generated from the significant regulators to classify patients into high- and low-score groups. In the training cohort, patients with high scores had shorter overall survival (HR, 5.19; 2.75–9.77; *P* < 0.001). The prognostic accuracy of the m^6^A score was well validated in two independent cohorts (HR 4.6, *P* = 0.006 and HR 3.07, *P* < 0.001). Time-dependent ROC and C-index analyses found the m^6^A score to possess superior predictive power than other clinicopathological parameters. A multicentre multivariate analysis revealed the m^6^A score to be an independent prognostic indicator. Additionally, patients with low scores received a greater survival benefit from ACT, exhibited more CD8+ T cell infiltration, and were more responsive to cancer immunotherapy.

**Conclusions:**

Our results, for the first time, affirm the significance of m^6^A regulators in LS-SCLC. Our multicentre analysis found that the m^6^A score was a reliable prognostic tool for guiding chemotherapy and immunotherapy selections for patients with SCLC.

**Supplementary Information:**

The online version contains supplementary material available at 10.1186/s12916-021-02148-5.

## Background

Small cell lung cancer (SCLC) is the most lethal high-grade neuroendocrine malignancy and features fast growth, early metastasis, and drug resistance. SCLC accounts for about 15% of all lung cancers; however, it has the highest mortality and worst outcomes—with a 5-year survival of < 7% [1, 2]. Regrettably, therapeutic strategies for SCLC have not significantly improved over recent decades. Conventional platinum-based chemotherapy remains the first-line treatment for patients with SCLC. Meanwhile, there have been few improvements in our ability to combat chemotherapy resistance for patients with SCLC [3]. Given the favourable achievements of immune checkpoint blockade (ICB) therapy for various tumours, this type of immunotherapy may be useful for SCLC treatment [4, 5]. Notably, a significant proportion of patients with ICB therapy resistance cannot benefit from such novel treatment [6–8]. Because of this, accurate and timely screening for patients who are more likely to benefit from immunotherapy is important.

PD-L1 expression is a classical biomarker for immunotherapy in various tumours, which is usually low or absent in SCLC. Consequently, it may fail to function as an immunotherapeutic biomarker [9, 10]. Therefore, there is an urgent and unmet need for a reliable predictive biomarker to guide the clinical application of chemotherapy and immunotherapy in patients with SCLC.

Dysregulation of epigenetic modifications relates to progression and treatment resistance in SCLC [11]. *N*^6^-methyladenosine (m^6^A) is the most prevalent type of RNA modification in eukaryotic cells [12], is responsible for various RNA-related biological processes—including RNA decay, stabilization, translation, splicing, and exportation—and ultimately regulates target gene expression [13]. Modification of m^6^A is a dynamic, multi-layered, and reversible process regulated by m^6^A methyltransferases, demethylases, and binding proteins [14]. Aberrant expression of m^6^A regulators appears closely related to carcinogenesis, tumour development, and immunological abnormalities [15, 16]. Multiple studies have revealed that m^6^A dysregulation dramatically enhances chemotherapy resistance in various tumours [17, 18]. Moreover, some m^6^A regulators can affect the response to immunotherapy [19, 20]. Increasing evidence suggests that m^6^A regulators are promising prognostic biomarkers which help determine chemotherapy and immunotherapy resistance. As the relevant research continues, these regulators’ relevance to a variety of tumours has been gradually confirmed [21, 22]. However, to the best of our knowledge, almost nothing is known about the roles of these m^6^A regulators in SCLC.

We examined the expression profiles, molecular characteristics, and prognostic significance of m^6^A regulators in SCLC. As early screening for lung cancer continues, the proportion of patients with limited-stage SCLC (LS-SCLC) is expected to similarly increase. We examined 265 cases with LS-SCLC from three independent cohorts and constructed an m^6^A regulator-based prognostic risk stratification score (m^6^A score) for patients with LS-SCLC. We additionally investigated the relationship between m^6^A score and adjuvant chemotherapy (ACT) benefit and response to anti-PD-1 treatment. Our findings may advance our ability to create individualized therapy regimens and guide SCLC prognostication.

## Methods

### Clinical samples and SCLC tissue specimens

We downloaded the training cohort somatic mutations and expression profiles (the international cohort) from Cbioportal (https://www.cbioportal.org/study/summary?id=sclc_ucologne_2015). During data processing, all RNA-seq data was subjected to log2 transformation. The mean expression values were selected when targeting genes that had more than one probe. We chose the GSE40275 database to explore the expression profile of m^6^A regulators, both in normal and LS-SCLC tissues. This dataset was downloaded from the Gene Expression Omnibus (GEO) dataset (https://www.ncbi.nlm.nih.gov/geo/) via the GPL15974 platform. Two validation cohorts—including the Shanghai cohort (GSE60052) downloaded from the GEO dataset and the National Cancer Centre (NCC) cohort, collected from the National Cancer Centre of China from January 2009 to November 2017—were collected. The NCC cohort included 150 LS-SCLC samples with formalin-fixed paraffin-embedded (FFPE) archived tissues were collected during surgeries. All enrolled patients from the NCC cohort were pathologically reconfirmed, had no other tumours, and carried clinically confirmed diagnoses of LS-SCLC. The relapse-free survival (RFS) and overall survival (OS) were defined as the day of surgical removal to recurrence, metastasis, or latest follow-up and the day of surgical removal to the date of death or latest follow-up. The study was approved by the Ethics Committee Board of our institute. The demographic and clinicopathologic parameters of the 150 LS-SCLC samples are displayed in Table 1.

**Table 1:** Clinical characteristics of the patients from multiple institutions

Characteristics	International cohort (*N* = 68)	Shanghai cohort (*N* = 47)	NCC cohort (*N* = 150)
Age, years			
< 60	16 (23.53%)	26 (55.32%)	81 (54.00%)
≥ 60	52 (76.47%)	21 (44.68%)	69 (46.00%)
Sex			
Male	48 (70.59%)	42 (89.36%)	119 (79.33%)
Female	20 (29.41%)	5 (10.64%)	31 (20.67%)
Smoking history			
Yes	64 (94.12%)	32 (68.09%)	94 (62.67%)
No	3 (4.41%)	15 (31.91%)	56 (37.33%)
NA	1 (1.47%)	0 (0.00%)	0 (0.00%)
SCLC staging			
I	33 (48.53%)	8 (17.02%)	49 (32.67%)
II	14 (20.59%)	39 (17.02%)	50 (33.33%)
III	21 (30.88%)	31 (65.96%)	51 (34.00%)
OS state			
Alive	28 (41.18%)	24 (51.06%)	69 (46.00%)
Death	40 (58.82%)	23 (48.94%)	81 (54.00%)

Data are *n* (%).

*NCC*, National Cancer Center; *NA*, not available; *SCLC*, small cell lung cancer; *OS*, overall survival

### Collection of samples with anti-PD-1 treatment

We included 14 patients with SCLC who received sequential chemotherapy and anti-PD-1 treatment in our hospital from April 2019 to January 2021. Their baseline biopsy FFPE specimens before immunotherapy were collected. The RECIST V1.1 Criteria were used to evaluate the response to therapy.

### Immunochemistry and quantification of CD8+ T cells

The 4-μm FFPE slides were subjected to immunochemical staining. After deparaffinization and rehydration with high-concentration ethanol and pure water, the slides were incubated in 3% H_2_O_2_ or 15 min to block endogenous peroxidase activity. Then, the slides were subjected to heat antigen retrieval and non-specific site blocking using an EDTA buffer (PD 9.0) and 10% standard serum, respectively. Next, the slides were incubated overnight at 4 °C. The final counterstaining was performed using secondary antibodies (CD8, Abcam, ab17147, 1:100) and the 3, 3′-diaminobenzidine (DAB, Dako, Glostrup, Denmark) and haematoxylin.

The digital pathological system (HALO) was utilized to quantify the density of CD8+ T cells on the whole slides. We scan the slides images at high resolution (× 400) using the Pannoramic MIDI II slide scanner (3DHISTECH). The tumour regions were identified by a trained pathologist (LYX). The “Membrane IHC Quantification” module was selected for absolute counting of CD8+ T cells on the CaseViewer_2.3.

### RNA isolation and quantitative RT-PCR

Only biopsies with at least 70% tumour cells were collected, and ~ 30-μm sections were cut from the FFPE samples. The Ambion RecoverAll Total Nucleic Acid Isolation Kit for FFPE (Thermo Fisher, Waltham, MA, USA) was used to isolate the FFPE tissue total RNA. The NanoDrop 2000C spectrophotometer (Thermo Scientific, Waltham, MA, USA) was used to quantify the extracted RNA. Meanwhile, the extracted RNA with an A260/A280 ratio between 1.8 and 2.2 were chosen for the quantitative RT-PCR (qRT-PCR) analysis. We used 200 ng RNA of a 20-μL reaction to reverse transcription through the FastKing Reverse Transcription Kit (Tiangen Biotech, Beijing, China). Next, we used 1 μL cDNA for PCR reaction with quantiNova PCR Kits (Qiagen, Dusseldorf, Germany) using 7900HT Fast Real-Time PCR system (Applied Biosystems, Carlsbad, USA; Indianapolis, IN). The qRT-PCR analysis was performed on all validation and independent cohort samples. The 2^−ΔΔCt^ method was used to calculate the expressions of interested m^6^A regulators. The details of the target m^6^A regulators primer sequences for qRT-PCR are shown in Additional file 1: Table S1.

### Construction of m^6^A regulator-based signature and statistical analysis

Firstly, we screened out the m^6^A regulators with prognostic significance based on the optimal cut-off point in the international cohort. We used the least shrinkage and selection operator (LASSO) Cox model to determine the most prognostic m^6^A regulators; the minimum criteria were chosen during the analysis process. Lastly, the final m^6^A score equation was accomplished based on the expression of the five chosen m^6^A regulators and corresponding Cox regression coefficients.

R version 3.5.1 (https://www.r-project.org) was used for all data analysis and image generation. The 30 m^6^A regulator protein-protein interaction analysis was conducted using the STRING interaction database and visualized using the Cytoscape software. The optimum cut-off survival analysis was completed using the “surv_cutpoint” function of the “survminer” R package. The Kaplan-Meier curve model was used to determine the prognostic value of the m^6^A regulator-based signature in the training and validation sets. The R package “survival” was employed to determine if the m^6^A score was an independent prognostic predictor for SCLC. The time-dependent receiver operating characteristic (ROC) curve analysis was created with the “survivalROC” R package. The Mann–Whitney *U* test was chosen to calculate the CD8+ T cell density between high- and low-score patients. A *P*-value less than 0.05 was considered statistically significant.

## Results

### Molecular characteristics of m^6^A regulators in SCLC

**Fig. 1:**
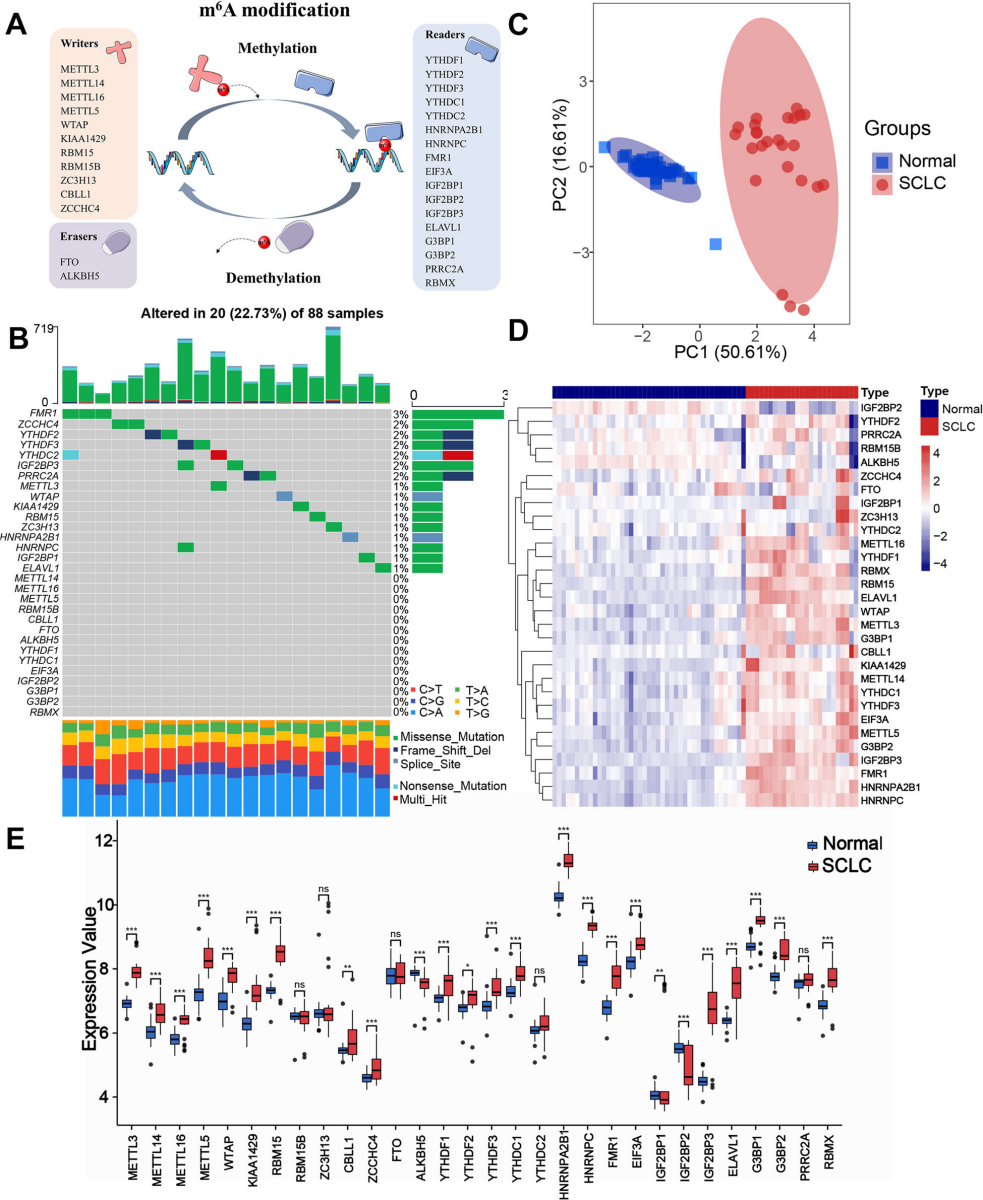
Molecular characteristics of m^6^A regulators in LS-SCLC. **A** Summary of the m^6^A regulators incorporated in this study. **B** The mutation frequency of m^6^A regulators in LS-SCLC. **C** Principal component analysis of SCLC and normal lung tissues based on the expression profile of 30 m^6^A regulators from GSE40275. **D** The heatmap of 30 m^6^A regulator expression from GSE40275. **E** The expression detail of m^6^A regulators in SCLC and normal lung tissues from GSE40275

After reviewing the latest relevant literature [23–26], we finally identified 30 m^6^A regulators, including 11 methyltransferases (METTL3, METTL14, METTL16, METTL5, WTAP, VIRMA, RBM15, RBM15B, ZC3H13, CBLL1, and ZCCHC4), 2 demethylases (ALKBH5 and FTO), and 17 binding proteins (YTHDF1, YTHDF2, YTHDF3, YTHDC1, YTHDC2, HNRNPA2B1, HNRNPC, FMR1, EIF3A, IGF2BP1, IGF2BP2, IGF2BP3, ELAVL1, G3BP1, G3BP2, PRRC2A, and RBMX) (Fig. 1A; Additional file 1: Table S2). Firstly, we explored the incidence of somatic mutations for m^6^A regulators in LS-SCLC. Mutations were present in 20 of 88 samples (22.73%; Fig. 1B). FMR1 displayed the highest mutation frequency, while approximately 14 m^6^A regulators exhibited no mutations within the LS-SCLC samples, including demethylases ALKBH5 and FTO (Fig. 1B). We identified co-occurrent mutations between METT3 and YTHDC2 and between HNRNPC and IGF2BP3 (Additional file 2: Fig. S1).

To determine if this genetic alteration affected the expression pattern of m^6^A regulators in LS-SCLC samples, we explored the mRNA expression of regulators between LS-SCLC and normal lung specimens. Principal component analysis revealed markedly different expression patterns of m^6^A regulators in LS-SCLC and normal samples (Fig. 1C). We also generated a heatmap for different expression profiles of these m^6^A regulators in LS-SCLC and normal tissues (Fig. 1D). The regulators’ expression details—between LS-SCLC and normal groups—are shown in Fig. 1E. Notably, almost all methyltransferases and binding proteins were upregulated in LS-SCLC; however, the two demethylases tended to exhibit lower expression in LS-SCLC than their normal counterparts. These results suggested that there may be abundant m^6^A modifications in LS-SCLC and significant heterogeneity in the m^6^A regulator genetic profile expression between LS-SCLC and normal lung tissues. Disordered m^6^A regulator expression may be involved in tumorigenesis and SCLC development.

### Association of various m^6^A regulators in SCLC

**Fig. 2:**
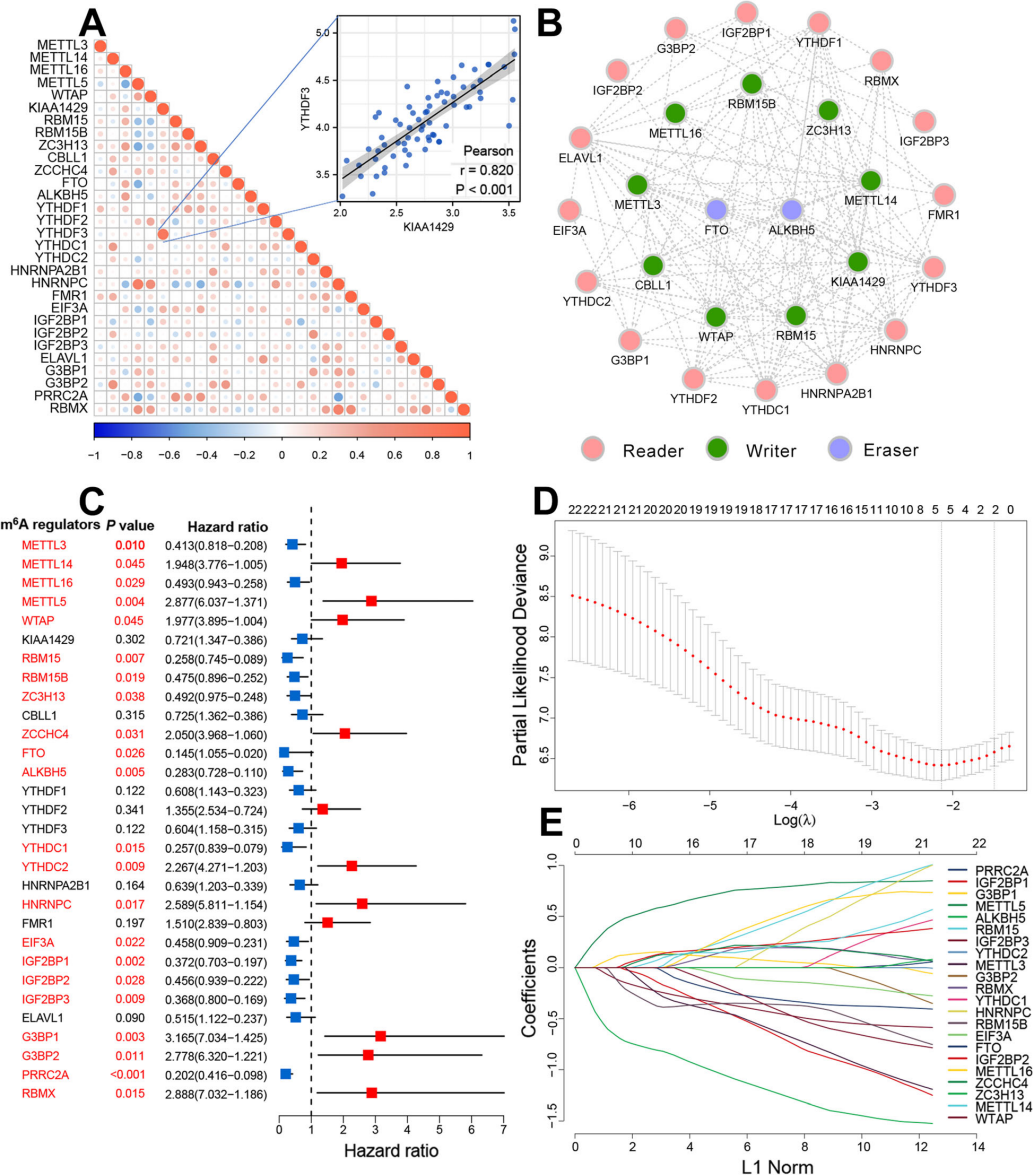
The clinical significance of m^6^A regulators in LS-SCLC. **A** Correlation between the expression of each m^6^A regulator in LS-SCLC. Negative correlations are marked with blue, and positive correlations are marked with red. The scatter plot indicates the highest correlation coefficient group (YTHDF3 and KIAA1429, Pearson *R* = 0.820). **B** Protein-protein interactions among the m^6^A regulators. **C** Forest plot of the association between m^6^A regulators and prognosis in SCLC. **D** 100-fold cross-validation for tuning parameter selection in the LASSO model. **E** LASSO coefficient profiles of the most useful prognostic regulators

Various m^6^A regulators cooperatively promote tumour development. Therefore, we also tried to explore the expression relationships for the 30 m^6^A regulators in SCLC. Notably, we detected remarkable correlations among the expressions of methyltransferases, demethylase, and binding proteins (Additional file 2: Fig. S2). Several significant positive correlations were also identified, including a correlation coefficient between KIAA1429 and YTHDF3 as high as 0.820 (Fig. 2A). Our protein-protein interaction network analysis determined that these 30 m^6^A regulators were in frequent communication (Fig. 2B). Importantly, the methyltransferases exhibited the most frequent interactions. Various methyltransferases formed a protein complex to perform biological functions. Therefore, the crosstalk among multiple m^6^A regulators may be actively involved in the SCLC development and progression.

### Clinical significance of m^6^A regulators in SCLC

Next, we investigated the clinical significance of m^6^A regulators in patients with LS-SCLC based on the optimal cut-off point derived from the international cohort. Most regulators were significantly associated with survival (Fig. 2C). Some regulators exhibited pro-carcinogenic properties, such as RBM15, RBM15B, ALKBH5, IGF2BP3, and PRRC2A. Some regulators functioned as tumour suppressors, including METTL5, YTHDC2, and G3BP1. Higher expression levels of these regulators often conveyed a favourable clinical prognosis. Given that most regulators exhibited clinical significance, we felt that an m^6^A regulator-based prognostic signature (m^6^A score) may be a useful molecular model for LS-SCLC. Therefore, using the LASSO Cox model, we included the above 22 regulators and determined five significant candidates—G3BP1, METTL5, ALKBH5, IGF2BP3, and RBM15B—for the subsequent m^6^A score creation (Fig. 2D, E).

### Establishment of the m^6^A score for LS-SCLC

**Fig. 3:**
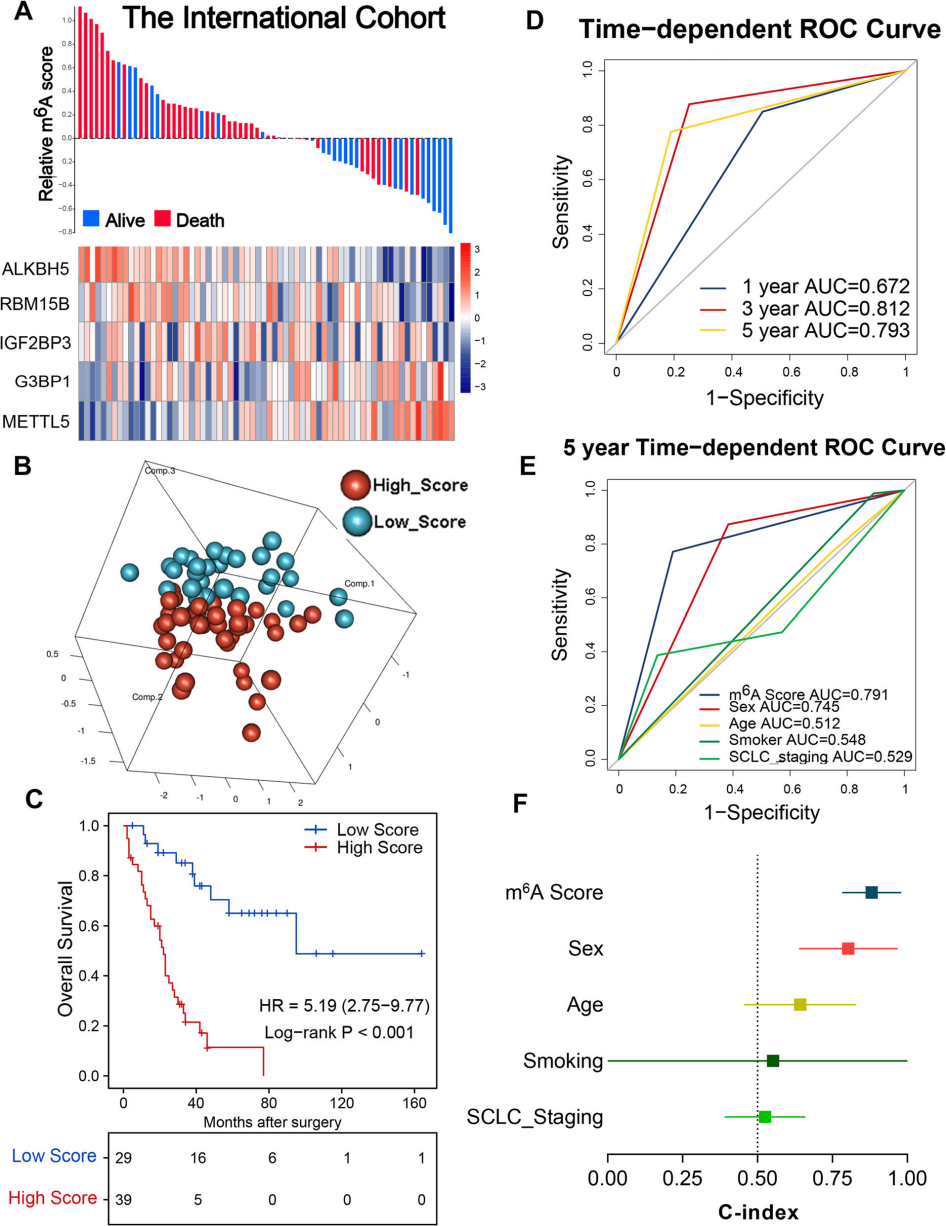
m^6^A score distribution and survival of patients in the international cohort. **A** m^6^A score distribution with patient survival status in the international cohort. The red colour indicates deceased patients while blue indicates survivors. Expression distribution of the five regulators in the international cohort, with red colour indicating higher expression and blue indicating lower expression. **B** Principal component analysis of high- and low-score patients based on the expression profile of five m^6^A regulators in the international cohort. **C** Kaplan-Meier curves of OS in 68 patients of the international cohort based on the m^6^A score. **D** ROC analysis of m^6^A score for the prediction of survival at 1, 3, and 5 years. **E** ROC analysis of m^6^A score and clinicopathological parameters for the prediction of survival at 5 years. **F** C-index values of m^6^A score and clinicopathological parameters for OS in the training cohort

Based on the expression levels of these five regulators and corresponding coefficients, we constructed the m^6^A score system for patients with LS-SCLC. The formula is as follows: m^6^A score = (0.0906 × *G3BP1* expression) + (0.4096 × *METTL5* expression) − (0.6365 × *ALKBH5* expression) − (0.0912 × *IGF2BP3* expression) − (0.0660 × *RBM15B* expression). We calculated the m^6^A scores for each patient and classified them into high- (m^6^A score ≥ − 1.271) and low-score (m^6^A score < − 1.271) groups according to the optimal cut-off point (Fig. 3A). The principal component analysis revealed high heterogeneity between the high- and low-score groups (Fig. 3B). The Kaplan-Meier survival curve results indicated that high-score patients suffered significantly worse OS (Fig. 3C, hazard ratios (HR) 5.19, 95% confidence interval (CI) 2.75–9.77, *P* < 0.001). To evaluate the prognostic performance of the m^6^A score, a time-dependent ROC analysis was conducted. The m^6^A score achieved area under the curve (AUC) values of 0.672, 0.812, and 0.793 for predicting OS within the international cohort at 1, 3, and 5 years, respectively (Fig. 3D). Further ROC analysis revealed that the m^6^A score performed better than clinicopathological characteristics for predicting OS (Fig. 3E). The C-index of the m^6^A score reached 0.881. This indicated that the prognostic accuracy of the m^6^A score was also higher than other clinicopathological parameters (Fig. 3F).

### Validation of the m^6^A score in multiple cohorts

**Fig. 4:**
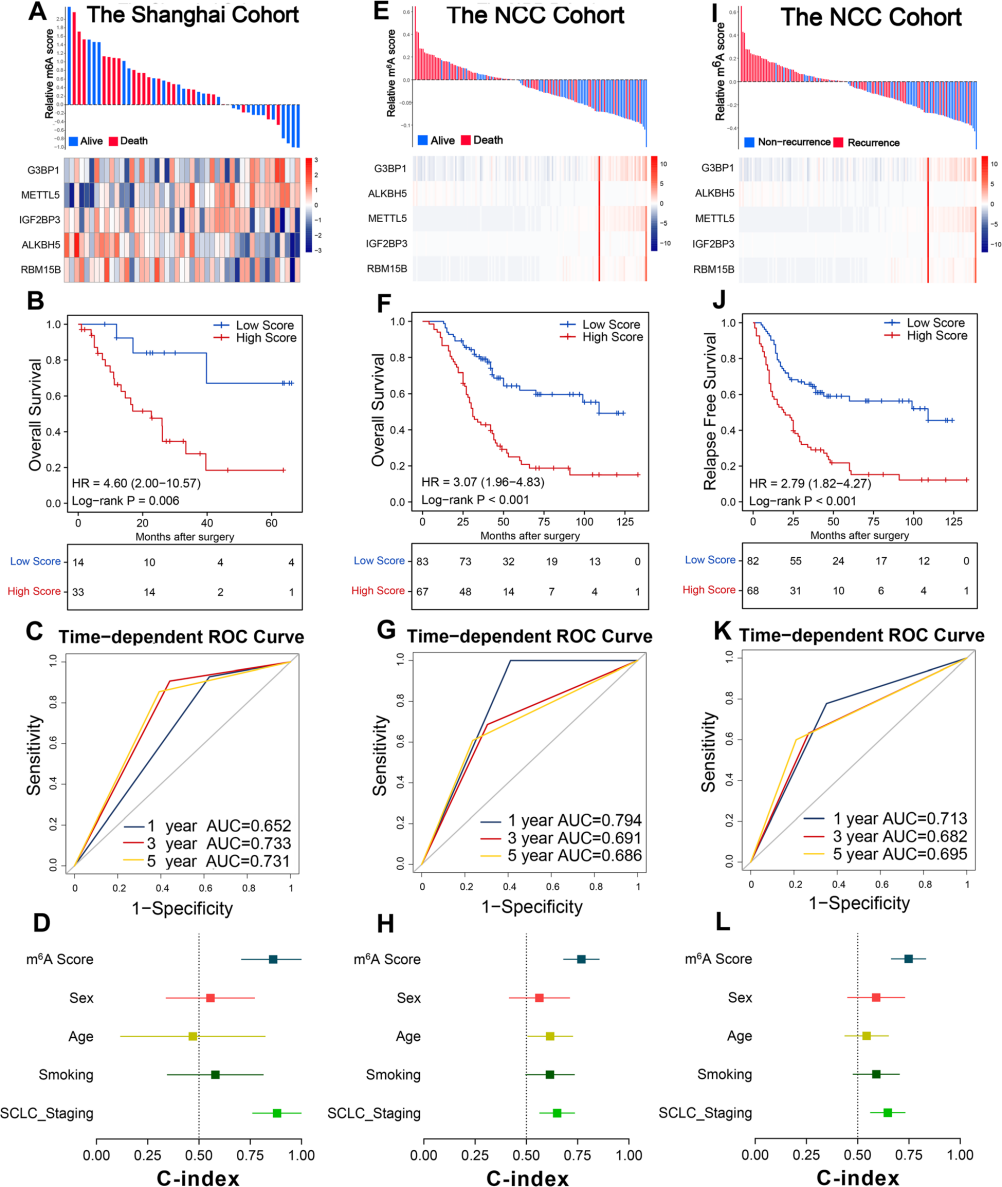
m^6^A score distribution and survival of patients in multiple validation cohorts. **A** The m^6^A score distribution with patient survival status in the Shanghai cohort. The red colour indicates deceased patients while blue indicates survivors. Expression distribution of the five regulators, with red colour indicating higher expression and blue indicating lower expression. **B** Kaplan-Meier curves of OS in 47 patients from the Shanghai cohort based on the m^6^A score. **C** ROC analysis of m^6^A score for survival prediction at 1, 3, and 5 years in the Shanghai cohort. **D** C-index values of the m^6^A score and clinicopathological parameters for OS in the Shanghai cohort. **E** The m^6^A score distribution with patient survival status in the NCC cohort. Expression distribution of the five regulators in the NCC cohort. **F** Kaplan-Meier curves of OS in 150 patients of the NCC cohort based on the m^6^A score. **G** ROC analysis of the m^6^A score for predicting survival at 1, 3, and 5 years in the NCC cohort. **H** C-index values of m^6^A score and clinicopathological parameters for OS in the NCC cohort. **I** The m^6^A score distribution with patient recurrence status in the NCC cohort, red indicating tumour recurrence while blue indicates no-recurrence. Expression distribution of the five regulators in the NCC cohort, with red colour indicating higher expression and blue indicating lower expression. **J** Kaplan-Meier curves of RFS in 150 patients of the NCC cohort based on m^6^A score. **K** ROC analysis of m^6^A score for the prediction of RFS at 1, 3, and 5 years in the NCC cohort. **H** C-index values of m^6^A score and clinicopathological parameters for RFS in the NCC cohort

To further assess the reliability and robustness of the classifier, we incorporated another two independent cohorts of 197 samples for validation, including the Shanghai cohort (*N* = 47) and the NCC cohort (*N* = 150). The cohorts’ clinicopathologic features are presented in Table 1. Using the same formula, the two cohorts were divided into low- and high-score groups. Firstly, we tested the m^6^A score in the Shanghai cohort. As expected, the signature showed excellent repeatability and stability during validation (Fig. 4A). The high-score patients in the Shanghai cohort suffered shorter OS than low-score patients (Fig. 4B, *P* = 0.006). The AUCs were 0.652, 0.733, and 0.731 for predicting 1-, 3-, and 5-year OS in the Shanghai cohort, respectively (Fig. 4C). In the Shanghai cohort, both the m^6^A score (C-index = 0.862) and SCLC staging (C-index = 0.880) accurately predicted OS for patients with LS-SCLC (Fig. 4D).

The clinical applicability of the m^6^A score was further evaluated in the FFPE specimens from the NCC cohort. Here, the low-score patients tended to significant better clinical outcomes in terms of OS (Fig. 4E, F, *P* < 0.001), and the m^6^A score achieved AUCs of 0.794, 0.691, 0.686 at 1-, 3-, and 5-year OS, respectively (Fig. 4G). Also, the C-index of the m^6^A score for OS was up to 0769 and higher than other factors in the NCC cohort (Fig. 4H).

We evaluated the predictive performance of the m^6^A score for RFS in patients with SCLC (Fig. 4I). The high m^6^A score was also predictive of poorer RFS in the NCC cohort (Fig. 4J, *P* < 0.001). The AUCs of m^6^A score for 1-, 3-, and 5-year RFS predictions were 0.713, 0.682, and 0.695, respectively, and the C-index was 0.748 in the NCC cohort (Fig. 4K, L). Thus, the m^6^A score was superior to the TNM system and sufficiently reliable to predict prognosis in patients with SCLC—both for OS and RFS.

We additionally explored the prognostic significance of the m^6^A score in relationship to various clinicopathological features—including sex, age, and smoking history. Because the sample size of the Shanghai cohort was small, we only performed clinical subgroup analyses on the international and NCC cohorts. As illustrated in Additional file 2: Fig. S3-S4, in the international cohort, low-score cases achieved longer OS and RFS across all clinical subtypes, including male, female, smoker, older (age ≥ 60), and younger (age < 60) (*P* < 0.05). The same results were obtained during the NCC cohort validation (Additional file 2: Fig. S3-S4, *P* < 0.05).

### The m^6^A score was an independent prognostic predictor in LS-SCLC

**Fig. 5:**
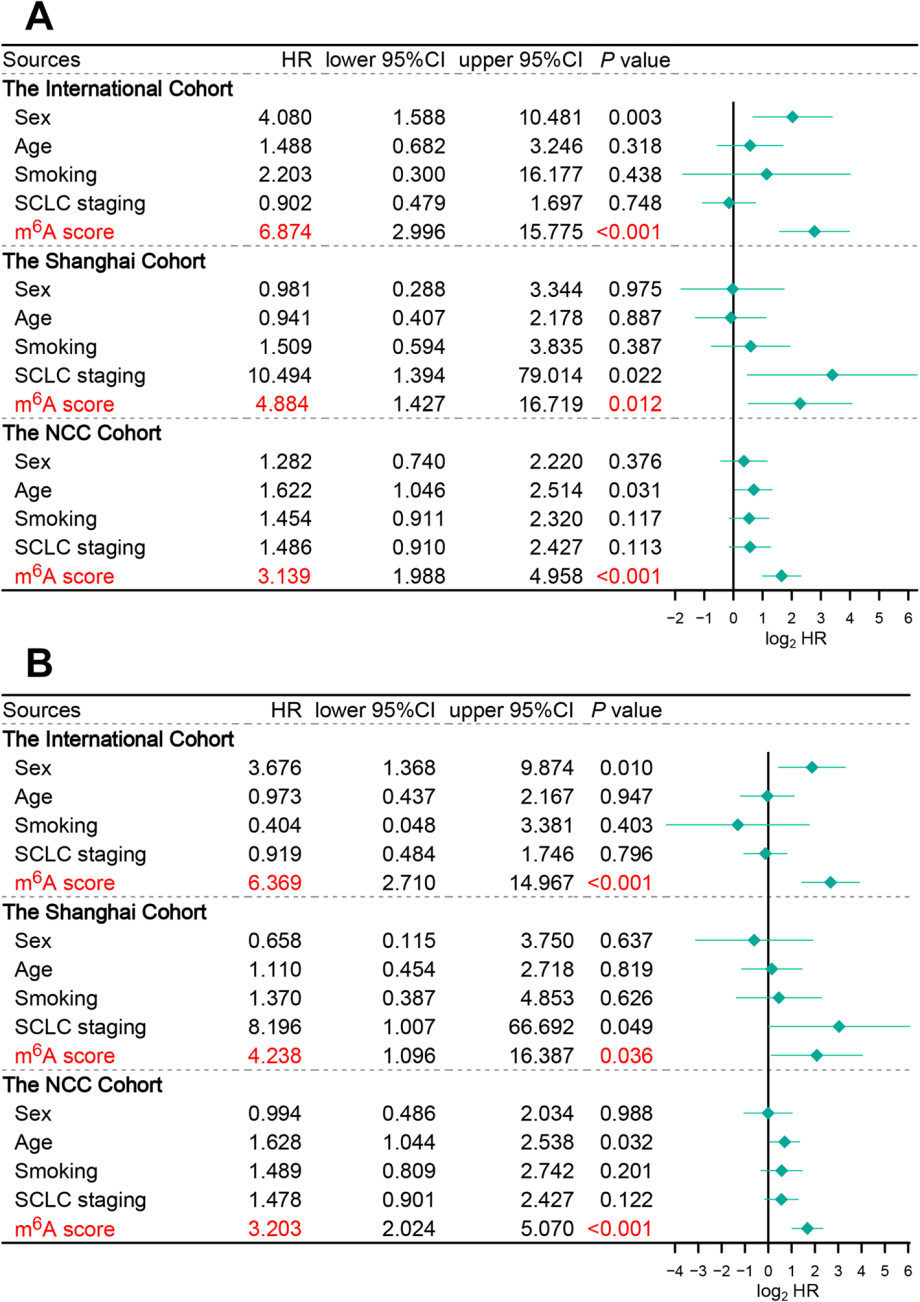
Cox regression analyses of the m^6^A score across multiple centres. **A** Univariate Cox regression analyses of m^6^A score and clinicopathological in three independent cohorts. **B** Multivariate Cox regression analyses of m^6^A score and clinicopathological in three independent cohorts

To confirm whether the m^6^A score is an independent predictor of prognosis in SCLC, we carried out univariate and multivariate Cox regression analyses on three independent cohorts. Sex and the m^6^A score were significantly related to OS in the international cohort; staging and the m^6^A score were also correlated with the prognosis in the Shanghai cohort. Age and the m^6^A score were significantly associated with survival in the NCC cohort (Fig. 5A, *P* < 0.05). Moreover, after integrating these clinical parameters into the multivariate Cox regression analyses, the m^6^A score was the only stable, independent prognostic indicator for patients with SCLC across all three cohorts (Fig. 5B, *P* < 0.05). Additionally, after multivariable adjustment by clinicopathological features, the m^6^A score remained a significant independent prognostic factor for RFS in the NCC cohort (Additional file 1: Table S3).

### The m^6^A score predicts the benefits of ACT

**Fig. 6:**
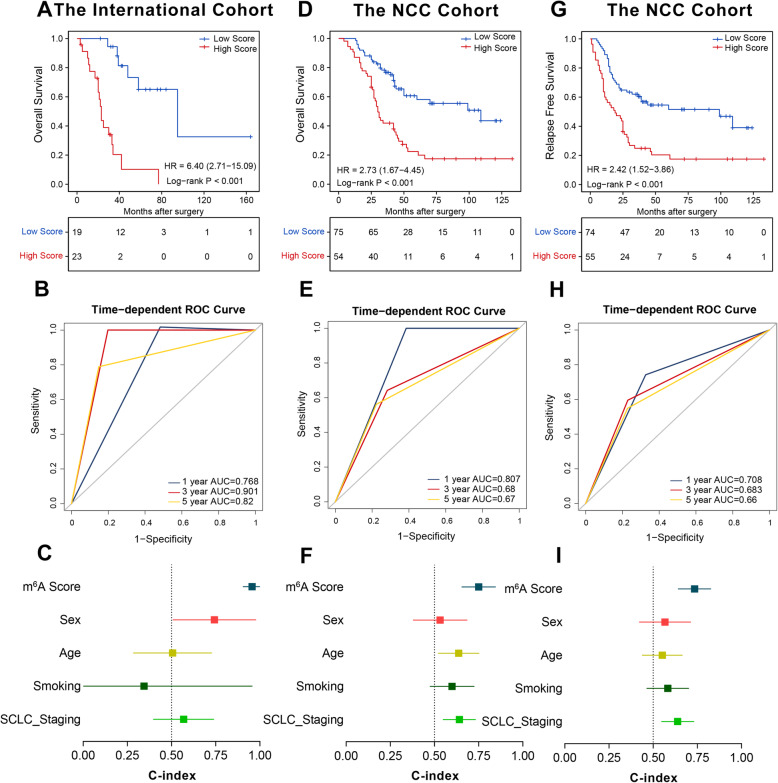
The predictive value of the m^6^A score for the benefit of adjuvant chemotherapy in different cohorts. **A** Kaplan-Meier curves of OS in patients with adjuvant chemotherapy in the international cohort. **B** ROC analysis of m^6^A score for the prediction of OS at 1, 3, and 5 years in patients with adjuvant chemotherapy in the international cohort. **C** C-index values of m^6^A score and clinicopathological parameters for OS in patients with adjuvant chemotherapy in the international cohort. **D** Kaplan-Meier curves of OS in patients with adjuvant chemotherapy in the NCC cohort. **E** ROC analysis of m^6^A score for predicting survival at 1, 3, and 5 years in patients with adjuvant chemotherapy in the NCC cohort. **F** C-index values of m^6^A score and clinicopathological parameters for OS in patients with adjuvant chemotherapy in the NCC cohort. **G** Kaplan-Meier curves of RFS in patients with adjuvant chemotherapy in the NCC cohort. **H** ROC analysis of m^6^A score for the prediction of RFS at 1, 3, and 5 years in patients with adjuvant chemotherapy in the NCC cohort. **I** C-index values of m^6^A score and clinicopathological parameters for RFS in patients with adjuvant chemotherapy in the NCC cohort

Considering the decisive role of m^6^A regulators in chemotherapy resistance, we further explored whether the m^6^A score could predict ACT treatment benefit. In the international and NCC cohorts, 42 and 129 cases underwent ACT, respectively. The m^6^A score divided 23 and 19 of 42 patients into high- and low-score groups in the international cohort (Fig. 6A), respectively, and divided 75 cases into the high-score group and 54 cases into the low-score group NCC cohort (Fig. 6D). Those low-score patients benefited considerably from ACT and achieved much longer OS than the high score cases in either cohort (Fig. 6A, D, both *P* < 0.001). Additionally, ROC curves showed that the AUCs of m^6^A score for predicting ACT OS benefit were 0.768, 0.901, and 0.82, and 0.807, 0.68, and 0.67 in the international cohort and NCC cohort for 1-, 3-, and 5-year, respectively (Fig. 6B, E). Meanwhile, the C-index of the m^6^A score for OS was also higher than other clinicopathological characteristics and as high as 0.956 and 0.750 in the two cohorts, respectively (Fig. 6C, F). In the NCC cohort, high-score cases suffered shorter RFS than the low-score ones (Fig. 6F, *P* < 0.001). The m^6^A score also achieved a reliable predictive ability to stratify different RFS statuses for patients with ACT. For AUCs of 0.708, 0.683, 0.66 at 1-, 3-, and 5-year RFS, the corresponding C-index was up to 0.734 (Fig. 6G, H). Collectively, the m^6^A score was able to identify those patients with SCLC most likely to benefit from ACT.

### Relationship between the m^6^A score and the anti-PD-1 immunotherapy response

**Fig. 7:**
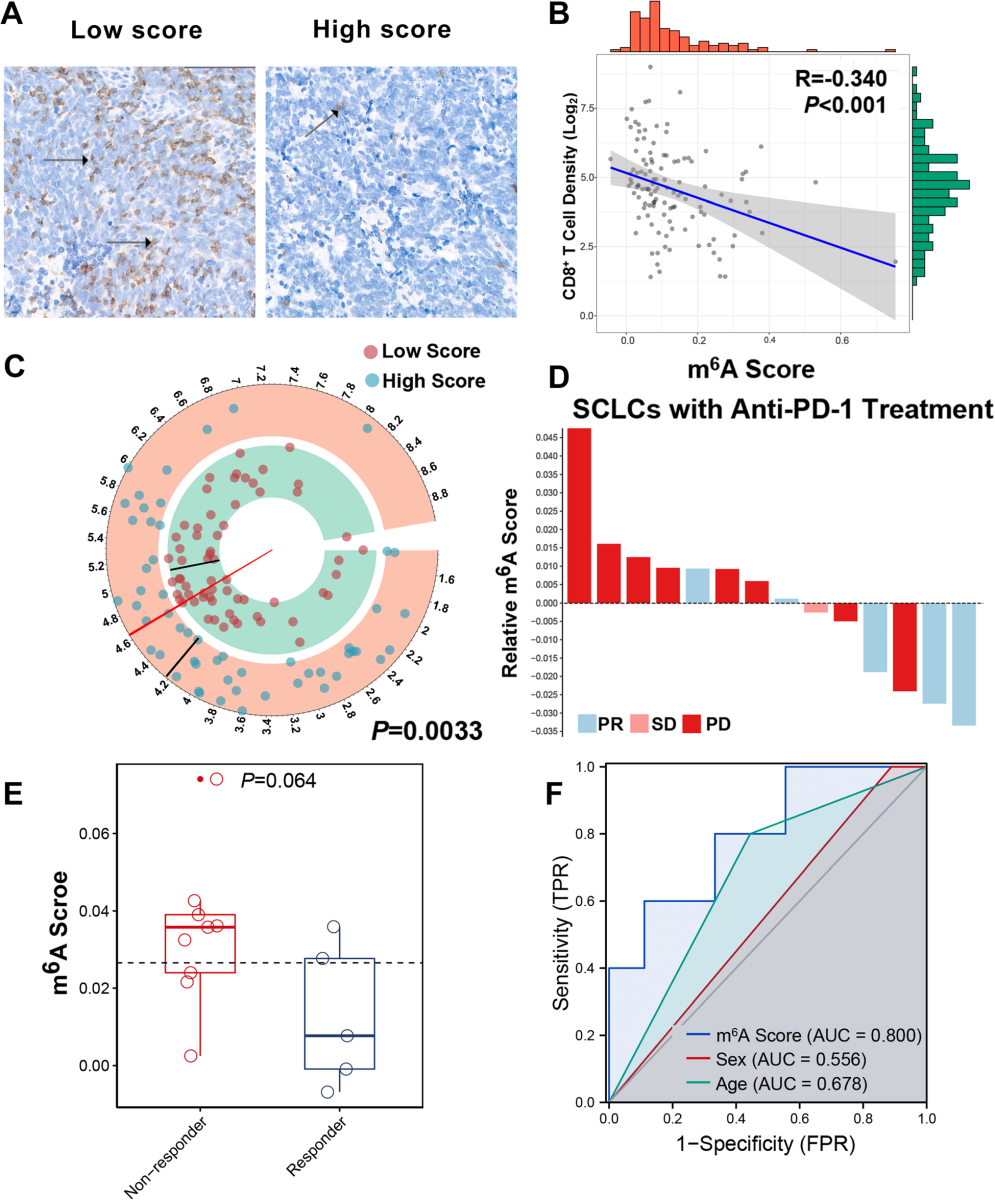
The m^6^A score predicts anti-PD-1 immunotherapy response in SCLC. **A** Representative immunohistochemical images of CD8+ T cell infiltration in SCLCs from the low- (case 1) and high-score (case 2) groups, respectively. The black arrows indicate the CD8 markers. **B** Pearson correlation analysis the CD8+ T cell density (log2 transformed) and the m^6^A score. **C** The density of CD8+ T cells (log2 transformed) in the low- and high-score groups. **D** A waterfall plot of m^6^A score distribution in patients with anti-PD-1 immunotherapy. Blue, pink, and red represent partial response (PR), stable disease (SD), and progressive disease (PD), respectively. **E** Differences of m^6^A scores in responders (PR) and non-responders (SD and PD). **F** ROC curves for the performance of the m^6^A score in predicting non-responders of immunotherapy in SCLC

Previous studies have demonstrated that m^6^A regulators relate to anti-tumour immune effects and tumour immune microenvironment (TIME) characterizations [27]. Since the m^6^A score is based on various m^6^A regulators, we decided to probe the relationship between the m^6^A signature and TIME features. Considering the centrality of CD8+ T cells in TIME, we explored the relationship between CD8+ T cell infiltration and the signature in SCLC. Using strict quality controls, we finally incorporated 117 FFPE samples in the NCC cohort. The density of CD8+ T cells in the tumour regions of SCLC was detected and calculated using the HALO digital pathological platform. Each patient’s m^6^A score was also matched. Representative pictures of CD8+ T cell distribution from high- and low-score groups are displayed in Fig. 7A. Low-score patients tended to have more CD8+ T cell infiltration than high-score patients (Fig. 7B). In addition, the m^6^A score was negatively correlated with CD8+ T cell density in SCLCs (Fig. 7C, *R* = − 0.34, *P* < 0.001).

We collected the pre-treatment samples from 14 patients with SCLC who received anti-PD-1 treatment to investigate the relationship between the m^6^A score and responses to immunotherapy. The overall response rate was 35.71%. Interestingly, patients with low scores seemed to benefit more from immunotherapy, while those with high scores tended to be resistant to immunotherapy (Fig. 7D, E). Meanwhile, ROC analyses indicated that the m^6^A score could predict non-responders with an AUC of 0.8. The m^6^A score showed superior performance than age or sex for identifying non-responders to immunotherapy (Fig. 7F).

## Discussion

Recent studies have indicated that m^6^A modification and multiple regulators play pivotal roles in tumorigenesis, tumour progression, and the anti-tumour immune response [11]. We also know that m^6^A regulators actively participate in mediating responses to chemotherapy and immunotherapy. Some proof-of-concept preclinical data have found that various m^6^A regulators inhibitors exhibit significant antitumor therapeutic potential, especially enabling dramatic increases in immunotherapy efficacy [19, 20, 28]. Therefore, the relevant mechanisms and clinical significance of m^6^A regulators are extremely important.

Although the functions of m^6^A modification and regulators in various tumours have been elucidated [21, 22], their roles and clinical values in SCLC were unknown. As our ability to detect and diagnose early-stage lung cancer increases, the proportion of LS-SCLC cases has similarly increased. We constructed an m^6^A regulator-based signature to predict prognosis for patients with LS-SCLC. We also explored the signature’s predictive role for chemotherapy and immunotherapy in SCLC. Our findings should enhance our understanding of tumorigenesis and help inform the clinical management of this disease.

Various epigenetic abnormalities are closely associated with the malignant phenotype, aggressiveness, metastasis, and therapeutic resistance of SCLC [11]. The m^6^A modification is the most essential RNA modification in eukaryotic cells; however, the m^6^A modification is poorly explored in SCLC. In the present study, we comprehensively revealed the m^6^A modification patterns in SCLC and identified that aberrant expression of m^6^A regulators was closely involved in SCLC tumorigenesis. We also found that most m^6^A methyltransferases and binding proteins were remarkably upregulated, while m^6^A demethylases were downregulated. Thus, abundant m^6^A modification may play a dominant role in SCLC progression.

We additionally excluded over 22 m^6^A regulators closely associated with SCLC prognosis and then established a five-regulator-based m^6^A score to effectively divide patients with SCLC into low- and high-score groups. During this process, the LASSO model was chosen because the collinearity relationships were found among the regulators. The low-score patients exhibited a more favourable prognosis than their high-score counterparts for OS and RFS. The signature was well-validated in various validation cohorts and was identified as an independent prognostic indicator for patients with SCLC. Moreover, we have also confirmed that our signature possesses significantly superior stratification ability for multiple clinical parameters among the three multicentre cohorts.

The m^6^A regulator-based signature included protective (ALKBH5, IGF2BP3, and RBM15B) and risk-enhancing (G3BP1 and METTL5) factors. ALKBH5, one of the classical m^6^A demethylases, decreases m^6^A modification in the target RNA. ALKBH5 is involved in the progression of multiple cancers, playing an oncogenic role in glioblastoma while suppressing the tumour proliferation and development in pancreatic cancer and NSCLC [29–31]. Meanwhile, a higher expression of ALKBH5 was also positively correlated with a favourable prognosis in gastric cancer; however, it was associated with worse clinical outcomes in colorectal cancer and NSCLC [32–34]. IGF2BP3 is a member of the IGF2 mRNA binding protein family—also known as the m^6^A binding protein—which exerts its biological functions in various human cancers [35]. IGF2BP3 functions as an oncofoetal factor in multiple tumour types, facilitating tumorigenesis by regulating the cell cycle, proliferation, and angiogenesis [36, 37]. In the previous studies, IGF2BP3 was considered a poor prognostic factor for NSCLC, prostate cancer, and endocrine system tumours [38–40]. RBM15B was classified into the m^6^A methyltransferases type, responsible for confirming that the m^6^A classical methyltransferase complex can function in specific regions. Higher expression levels of RBM15B tend to confer better clinical outcomes for patients with kidney renal cell carcinoma [41].

Among the risky candidates, G3BP1 was a novel m^6^A-binding protein that affects mRNA stability via an m^6^A modification manner. This further regulated some essential oncogenic signal pathways to promote tumour metastasis and aggressiveness [42]. The elevated expression of G3BP1 confers a worse prognosis for patients with lung cancer after surgery [43]. METTL5 is a novel m^6^A methyltransferase, mainly adding m^6^A modification for ribosomal RNA [44]. Our earlier work found that METTL5 was significantly associated with a worse prognosis in NSCLC [45]. One small-scale study sought to determine the function of METTL5 in carcinogenesis; however, additional studies are needed.

We could also use the m^6^A score to identify patients with SCLC who were more likely to benefit from ACT. Our novel m^6^A score possessed a better predictive capacity of ACT efficacy than TNM staging. This may be useful for the individualized application of ACT in patients with SCLC. Additionally, some m^6^A regulators in the signature appeared closely associated with chemotherapy resistance. ALKBH5 can induce cisplatin resistance by decreasing the m^6^A modification on the FOXM1 and NANOG transcripts and increasing their expression [46]. Also, upregulating ALKBH5 expression sensitizes pancreatic ductal adenocarcinoma cells to chemotherapy treatment, indicating that ALKBH5 may play the same role in SCLC [30]. Chen et al. reported that IGF2BP3 sustained the pluripotency in hepatocellular carcinoma (HCC) cells and triggered chemoresistance in HCC [47]. Lower expression of G3BP1 increases the chemotherapy sensitivity in gastric cancer cells and predicts favourable benefits of chemotherapy and prognosis for patients with gastric cancer. This is in accordance with the potential role of G3BP1 in our m^6^A score system in SCLC [48]. Collectively, we speculate that the regulators in the m^6^A score may help regulate ACT sensitivity and resistance in SCLC. Future studies are necessary to uncover the underlying relationships between these regulators and chemotherapy resistance in SCLC.

We discovered a relationship between the m^6^A score and immunotherapy responses in SCLC. PD-L1 expression and CD8+ T cells are closely associated with the efficacy of immunotherapy on various malignancies. Notably, PD-L1 expression is typically low or absent in SCLC. Given the obvious subjectivity and uncertainty in interpreting PD-L1 expression, we finally explored the relationship between CD8+ T cells and m^6^A score in SCLC [49]. As expected, the m^6^A score was closely correlated with CD8+ T cells in SCLC, and patients with low scores exhibited higher CD8+ T infiltration.

Then, we investigated the potential role of the m^6^A score in predicting the immunotherapy response in patients with SCLC. Consistent with the above observations, low-score patients were more likely to benefit from immunotherapy. We also noted that some signature members appeared to relate to immunotherapy efficacy, especially demethylase ALKBH5. ALKBH5 regulates the immunotherapy responses by manipulating the accumulation of suppressive immune cells in TIME, actively modulating the infiltration of Tregs and myeloid-derived suppressor cells [50]. ALKBH5 may participate in the composition and function of CD8+ T cells in the TIME, ultimately affecting the response to immunotherapy in SCLC, while other regulators may also function in the same way. Further exploring the functions of these five m^6^A regulators may help us understand the nature of SCLC and provide some clues to further personalize immunotherapy application in patients with SCLC.

To our best knowledge, this is the first systematic examination of m^6^A modification patterns in LS-SCLC. We established a comprehensive m^6^A regulator prognostic signature based on data obtained from over 265 patients with LS-SCLC from three centres. Large-scale retrospective SCLC analyses are exceptionally rare due to challenges in obtaining available tumour specimens within the context of standardized treatment regimens.

Our innovative signature has certain advantages. Firstly, the large size of our study cohort increases the reliability and robustness of our model. Additionally, our signature is the first molecular model to predict chemotherapy and immunotherapy efficacy for patients with SCLC based on tissue samples. This signature may therefore be useful in treating and clinically managing patients with SCLC.

In addition to these advantages, our study also possesses some limitations which warrant consideration. Firstly, we validated the NCC cohort using retrospective FFPE specimens. Future, studies should collect and examine fresh specimens in a prospective manner. Secondly, despite we did our best efforts to collect the immunotherapy samples for validation, we only included 14 patients with SCLC who received immunotherapy. This is likely insufficient for conducting a comprehensive analysis. Thirdly, given that this was a retrospective study, there is likely to be unavoidable bias and error in the analysis. Prospective, well-powered studies are needed to further validate the reliability of the signature.

## Conclusions

In conclusion, we demonstrated the significance of m^6^A modification in SCLC and developed the first and most comprehensive multicentre m^6^A regulator-based prognostic signature for patients with LS-SCLC. This m^6^A signature can accurately predict OS, RFS, chemotherapy benefit, and immunotherapy response in patients with SCLC. The m^6^A signature can therefore serve as both a prognostic and predictive tool for SCLC. Further prospective validation of the predictive ability of the m^6^A score will aid our ability to effectively treat patients with SCLC.

## Supplementary Information

**Additional file 1: Table S1.** Primer sequences of the samples from the NCC cohort for qPCR. **Table S2.** The descriptions of the 30 m^6^A regulators collected in this study. **Table S3.** Univariable and multivariate Cox regression of m^6^A score and clinicopathological characteristics and relapse free survival in SCLC.

**Additional file 2: Fig. S1.** Co-occurrence of genetic alterations of the m6A regulators in SCLC. **Fig. S2.** Correlation between the expression of m6A regulators in SCLC. **Fig. S3.** Validation of the OS predictive performance of the m6A score across clinical subgroups. **Fig. S4.** Validation of the RFS predictive performance of the m6A score across clinical subgroups.

## Data Availability

The datasets used and analysed during the current study are available from the corresponding author on reasonable request.

## Abbreviations

ACT: Adjuvant chemotherapy
AUC: Area under the curve
CI: Confidence interval
FFPE: Formalin-fixed paraffin-embedded
GEO: Gene Expression Omnibus
HCC: Hepatocellular carcinomas
HR: Hazard ratios
ICB: Immune checkpoint blockade
LASSO: Least shrinkage and selection operator
LS-SCLC: limited-stage SCLC
m^6^A: *N*^6^-methyladenosine
NCC: National Cancer Centre
OS: Overall survival
RFS: Relapse-free survival
ROC: Receiver operating characteristic
SCLC: Small cell lung cancer
TIME: Tumour immune microenvironment
