# Supplementary material for: m6A regulator expression profile predicts the prognosis, benefit of adjuvant chemotherapy, and response to anti-PD-1 immunotherapy in patients with small-cell lung cancer
Source: BMC Med. 2021 Nov 22;19:284. doi: 10.1186/s12916-021-02148-5 (PMC8607595; doi:10.1186/s12916-021-02148-5)
Supplement: Supplementary file 1 — Additional file 1: Table S1. Primer sequences of the samples from the NCC cohort for qPCR. Table S2. The descriptions of the 30 m6A regulators collected in this study. Table S3. Univariable and multivariate Cox regression of m6A score and clinicopathological characteristics and relapse free survival in SCLC. [file 12916_2021_2148_MOESM1_ESM.docx]

**Table S1**. Primer sequences of the samples from the NCC cohort for qPCR.

| Gene Name | Forward Primer | Reverse Primer |
| --- | --- | --- |
| G3BP1 | 5'-CACAAAGACCTCAGCGGGAT-3' | 5'-CTCACGGATTGGTCTGGGTC-3' |
| METTL5 | 5'-GTACGCGGAGTGGCAGAAA-3' | 5'-CAGAGGACGTTGCAGTAGC-3' |
| ALKBH5 | 5'-TCAAGCCTATTCGGGTGTCG-3' | 5'-ATCCACTGAGCACAGTCACG-3' |
| IGF2BP3 | 5'-TGCCACCATTCGGAACATCA-3' | 5'-AATCGACTTCTCAGCAGCCC-3' |
| RBM15B | 5'-GGGAGCATTCGGACCATTGA-3' | 5'-CTCATTTTAGCACAGGCGGC-3' |
| GAPDH | 5'-AAATCAAGTGGGGCGATGCT-3' | 5'-CAAATGAGCCCCAGCCTTCT-3' |

**Table S2.** The descriptions of the 30 m^6^A regulators collected in this study.

| Category | Official symbol | Gene ID | Description |
| --- | --- | --- | --- |
| Writer | METTL3 | 56339 | Methyltransferase like 3 |
| Writer | METTL14 | 57721 | Methyltransferase like 14 |
| Writer | METTL16 | 79066 | Methyltransferase like 16 |
| Writer | METTL5 | 29081 | Methyltransferase like 5 |
| Writer | WTAP | 9589 | WT1 associated protein |
| Writer | VIRMA | 25962 | Vir like m6A methyltransferase associated |
| Writer | RBM15 | 64783 | RNA binding motif protein 15 |
| Writer | RBM15B | 29890 | RNA binding motif protein 15B |
| Writer | ZC3H13 | 23091 | Zinc finger CCCH-type containing 13 |
| Writer | CBLL1 | 79872 | Cbl proto-oncogene like 1 |
| Writer | ZCCHC4 | 29063 | Zinc finger CCHC-type containing 4 |
| Reader | YTHDF1 | 54915 | YTH N6-methyladenosine RNA binding protein 1 |
| Reader | YTHDF2 | 51441 | YTH N6-methyladenosine RNA binding protein 2 |
| Reader | YTHDF3 | 253943 | YTH N6-methyladenosine RNA binding protein 3 |
| Reader | YTHDC1 | 91746 | YTH domain containing 1 |
| Reader | YTHDC2 | 64848 | YTH domain containing 2 |
| Reader | HNRNPA2B1 | 3181 | Heterogeneous nuclear ribonucleoprotein A2/B1 |
| Reader | HNRNPC | 3183 | Heterogeneous nuclear ribonucleoprotein C |
| Reader | FMR1 | 2332 | FMRP translational regulator 1 |
| Reader | EIF3A | 8661 | Eukaryotic translation initiation factor 3 subunit A |
| Reader | IGF2BP1 | 10642 | Insulin like growth factor 2 mRNA binding protein 1 |
| Reader | IGF2BP2 | 10644 | Insulin like growth factor 2 mRNA binding protein 2 |
| Reader | IGF2BP3 | 10643 | Insulin like growth factor 2 mRNA binding protein 3 |
| Reader | ELAVL1 | 1994 | ELAV like RNA binding protein 1 |
| Reader | G3BP1 | 10146 | G3BP stress granule assembly factor 1 |
| Reader | G3BP2 | 9908 | G3BP stress granule assembly factor 2 |
| Reader | PRRC2A | 7916 | Proline rich coiled-coil 2A |
| Reader | RBMX | 27316 | RNA binding motif protein X-linked |
| Eraser | FTO | 79068 | FTO alpha-ketoglutarate dependent dioxygenase |
| Eraser | ALKBH5 | 54890 | AlkB homolog 5, RNA demethylase |

**Table S3**. Univariable and multivariate Cox regression of m^6^A score and clinicopathological characteristics and relapse free survival in SCLC

| Variable |  | Univariate | |  | Multivariate | |
| --- | --- | --- | --- | --- | --- | --- |
|  |  | HR  (95% CI) | *P* value |  | HR  (95% CI) | *P* value |
| Sex  Male/Female |  | 1.500  (0.872-2.581） | 0.143 |  | 1.177  (0.593-2.336) | 0.642 |
| Age  ≥60/<60 |  | 1.191  (0.787-1.800) | 0.408 |  | 1.214  (0.797-1.850) | 0.366 |
| Smoking  Smoking/Non-smoking |  | 1.456  (0.936-2.266) | 0.096 |  | 1.345  (0.764-2.366) | 0.305 |
| SCLC staging  II, III/I |  | 1.724  (1.073-2.771) | 0.024 |  | 1.577  (0.977-2.546) | 0.062 |
| m^6^A score  High/Low |  | 2.746  (1.801-4.189) | <0.001 |  | 2.696  (1.763-4.122) | <0.001 |

Abbreviations: SCLC, small cell lung cancer; HR, hazard ratio; CI, confidence interval.
