# Supplementary material for: m6A regulator expression profile predicts the prognosis, benefit of adjuvant chemotherapy, and response to anti-PD-1 immunotherapy in patients with small-cell lung cancer
Source: BMC Med. 2021 Nov 22;19:284. doi: 10.1186/s12916-021-02148-5 (PMC8607595; doi:10.1186/s12916-021-02148-5)

**Figure S1. Co-occurrence of genetic alterations of the m^6^A regulators in SCLC.**


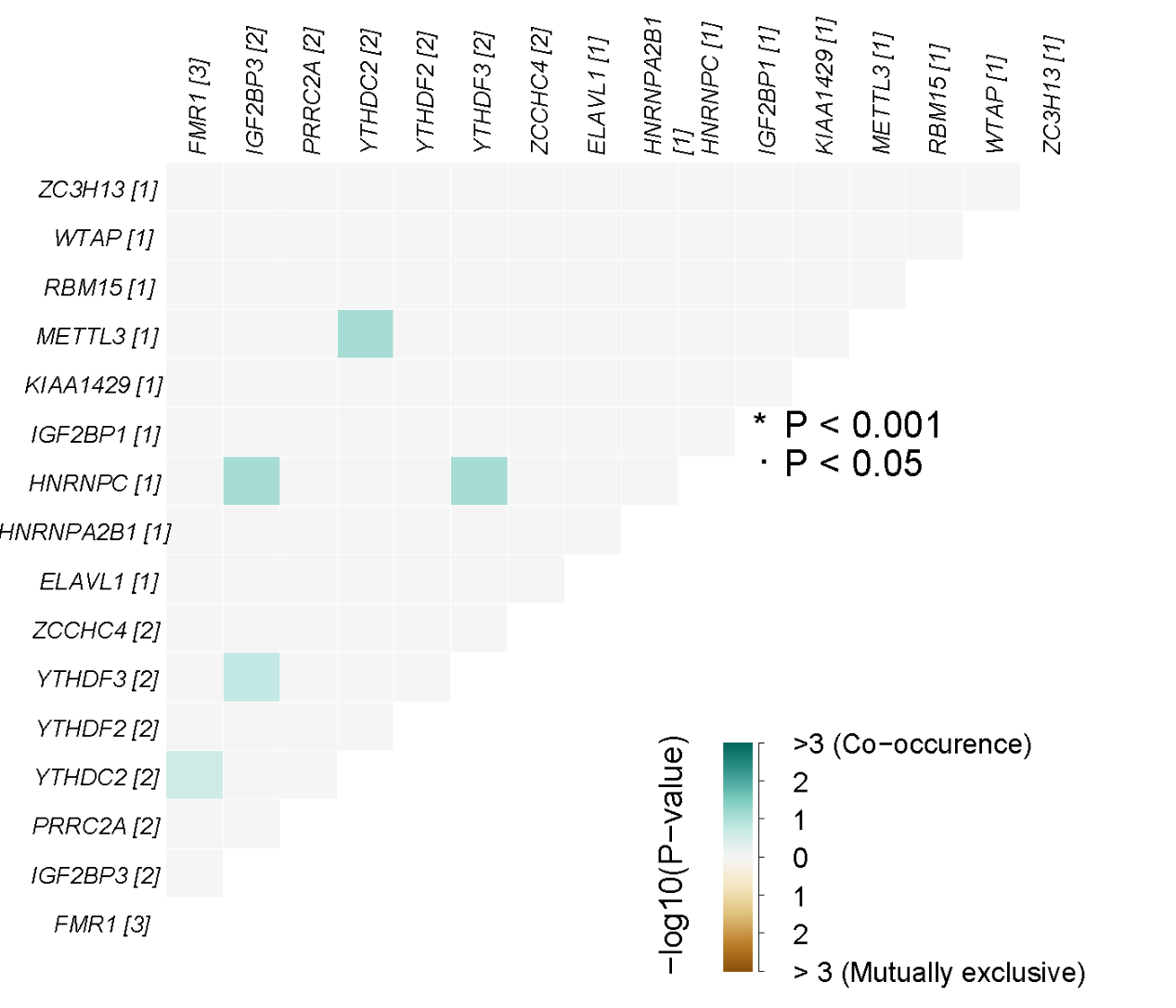


**Figure S2. Correlation between the expression of m^6^A regulators in SCLC.** (A) The scatter plot indicates the significant correlation coefficient group (HNRNPC and METTL5, Pearson R= 0.750). (B) The scatter plot indicates the significant correlation coefficient group (HNRNPC and WTAP, Pearson R= 0.600). (C) The scatter plot indicates the significant correlation coefficient group (IGF2BP2 and YTHDC2, Pearson R= 0.510). (D) The scatter plot indicates the significant correlation coefficient group (G3BP2 and METTL14, Pearson R= 0.550). (E) The scatter plot indicates the significant correlation coefficient group (PPRC2A and RBM15, Pearson R= 0.530). (F) The scatter plot indicates the significant correlation coefficient group (PPRC2A and ZC3H14, Pearson R= 0.510). (G) The scatter plot indicates the significant correlation coefficient group (PPRC2A and HNRNPC, Pearson R= - 0.510). (H) The scatter plot indicates the significant correlation coefficient group (RBMX and METTL5, Pearson R= 0.520). (I) The scatter plot indicates the significant correlation coefficient group (RBMX and HNRNPC, Pearson R= 0.710). (J) The scatter plot indicates the significant correlation coefficient group (RBMX and FMR1, Pearson R= 0.600). (K) The scatter plot indicates the significant correlation coefficient group (RBMX and G3BP1, Pearson R= 0.550).


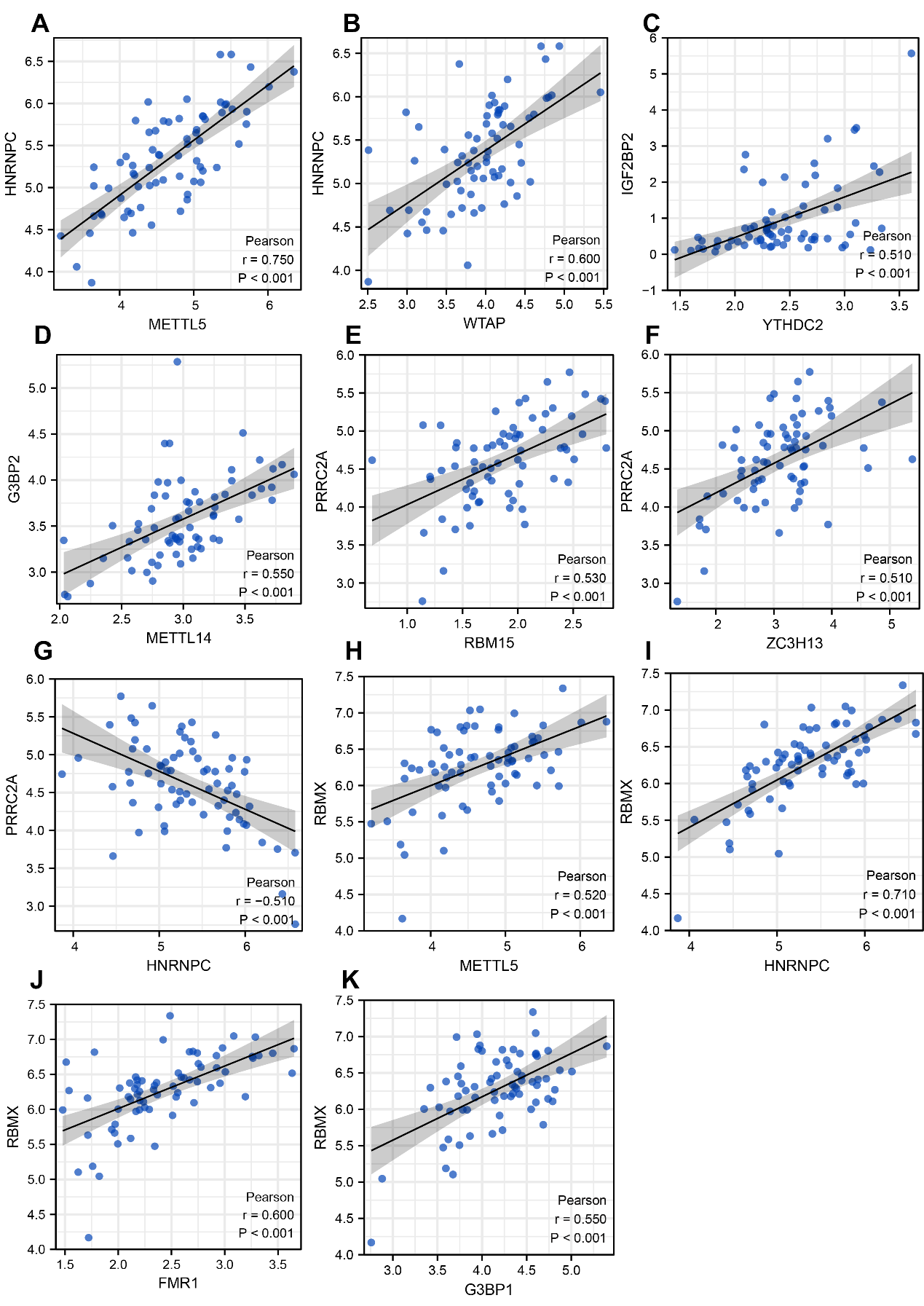


**Figure S3.** **Validation of the OS predictive performance of the m^6^A score across clinical subgroups.** (A) Kaplan-Meier curves of OS in training cohort males. (B) Kaplan-Meier curves of OS in NCC cohort males. (C) Kaplan-Meier curves of OS in the training cohort females. (D) Kaplan-Meier curves of OS in NCC cohort males. (E) Kaplan-Meier curves of OS in the smoker of the training cohort. (F) Kaplan-Meier curves of OS in the male of the NCC cohort. (G) Kaplan-Meier curves of OS in non-smoker of the NCC cohort. (H) Kaplan-Meier curves of OS in older of the training cohort. (I) Kaplan-Meier curves of OS in older of the NCC cohort. (J) Kaplan-Meier curves of OS in young of the training cohort. (K) Kaplan-Meier curves of OS in young of the NCC cohort.


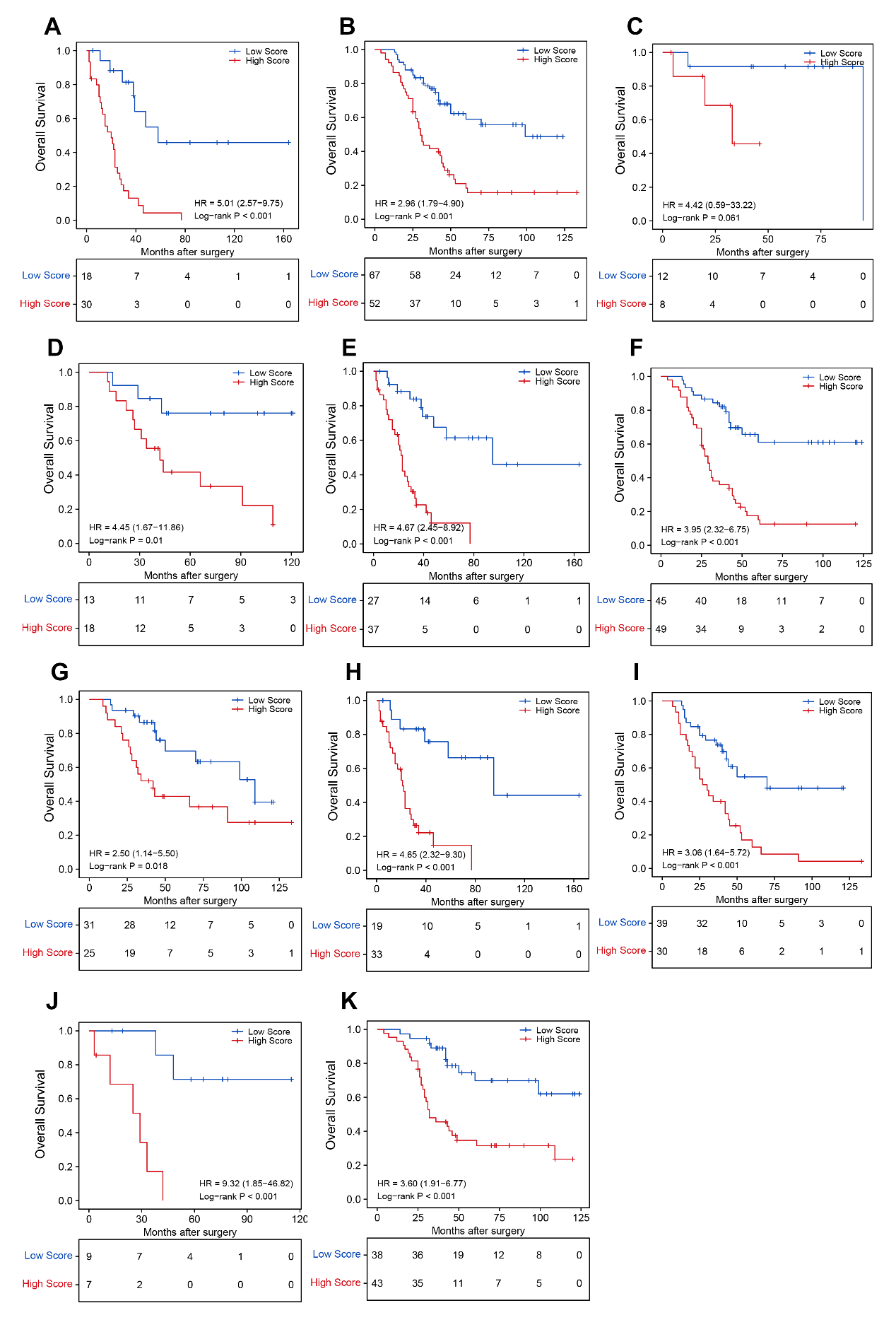


**Figure S4. Validation of the RFS predictive performance of the m^6^A score across clinical subgroups.** (A) Kaplan-Meier curves of RFS in the male of the NCC cohort. (B) Kaplan-Meier curves of RFS in the female of the NCC cohort. (C) Kaplan-Meier curves of RFS in the smoker of the NCC cohort. (D) Kaplan-Meier curves of RFS in non-smoker of the NCC cohort. (E) Kaplan-Meier curves of RFS in older of the NCC cohort. (F) Kaplan-Meier curves of RFS in young of the NCC cohort**.**


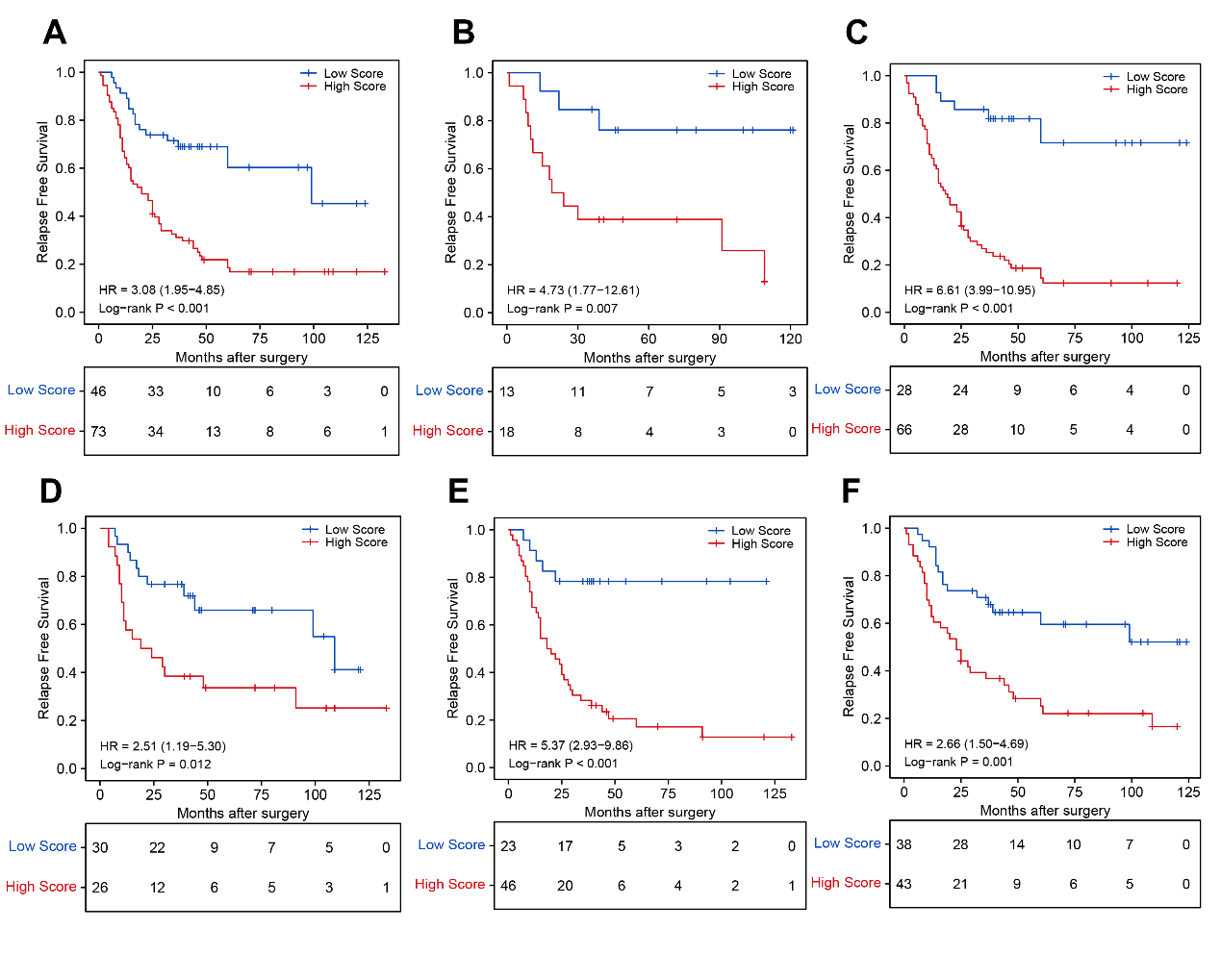

Supplement: Supplementary file 2 — Additional file 2: Fig. S1. Co-occurrence of genetic alterations of the m6A regulators in SCLC. Fig. S2. Correlation between the expression of m6A regulators in SCLC. Fig. S3. Validation of the OS predictive performance of the m6A score across clinical subgroups. Fig. S4. Validation of the RFS predictive performance of the m6A score across clinical subgroups. [file 12916_2021_2148_MOESM2_ESM.docx]
